# Efficacy of 0.05% cyclosporine A on tear inflammatory cytokines and goblet cell function after corneal refractive surgery

**DOI:** 10.1186/s12348-025-00462-0

**Published:** 2025-04-03

**Authors:** Wenzhe Qian, Yue Wu, Xin Liu, Yuying Liu, Min Li, Ting Zhao, Na Chen, Bilian Ke

**Affiliations:** 1 Department of Ophthalmology, Shanghai General Hospital, Shanghai Jiao Tong University School of Medicine, No. 100 Haining Road, Shanghai, 200080 China; 2 National Clinical Research Center for Eye Diseases, Shanghai, China; 3 Department of Ophthalmology, Shanghai Renji Hospital, Shanghai Jiao Tong University School of Medicine, No. 160 Pujian Road, Shanghai, 200127 China

**Keywords:** Cyclosporine A, Refractive surgery, Tear film, Interferon-γ, Tumor Necrosis Factor-α, Conjunctival epithelium, Goblet cell, Mucin 5AC

## Abstract

**Background:**

Corneal refractive surgeries offer rapid vision correction, while dry eye disease remains a common postoperative complication that significantly impacts patients' quality of life. The etiology of postoperative dry eye is multifactorial. Cyclosporine A (CsA) has been employed in the treatment of dry eye due to its ability to suppress T cell-mediated immune responses and reduce inflammation. The present study was designed to assess the early effect of 0.05% cyclosporine A (CsA) eye drops on tear film stability, ocular surface inflammation and goblet cell function in patients following corneal refractive surgery.

**Methods:**

One hundred forty-four eyes of 72 participants undergoing corneal refractive surgery were enrolled and randomized into additional 0.05% CsA eye drops treated group or conventional schedule treated group. Ocular Surface Disease Index (OSDI), relevant ocular surface clinical parameters, tear inflammatory cytokine levels, conjunctival impression cytology, and gene expression of Keratin 7 (KRT-7) as well as Mucin5AC (Muc5AC) in conjunctival epithelial cells were measured before surgery (baseline) and at 1 month after surgery. All indicators and their changing value were compared against baseline or across different groups.

**Results:**

0.05% CsA treatment exhibited greater changes in OSDI, NIBUT, LLT and CFS in the early postoperative period (*P* = 0.004, *P* = 0.002, *P* = 0.032, *P* = 0.008). Compared to control group, there was a more significant decrease in IFN-γ and TNF-α levels in tear fluid in CsA group after surgery (*P* = 0.012, *p* = 0.032). Additionally, KRT-7 and IFN-γ showed recovery in conjunctival cells with 0.05% CsA treatment (*P* = 0.003, *P* = 0.019). The postoperative KRT-7 and Muc5AC levels were negatively correlated with corresponding IFN-γ levels in tear fluid among all subjects (r = -0.200, *p* = 0.016; r = -0.229, *p* = 0.006).

**Conclusions:**

For patients undergoing refractive surgery, the application of 0.05% CsA suppressed the expression of inflammatory cytokines such as IFN-γ and TNF-α, and preserved goblet cell function. These effects ultimately contribute to maintaining ocular surface stability and alleviating dry eye related symptoms during the early postoperative period following refractive surgery.

**Supplementary Information:**

The online version contains supplementary material available at 10.1186/s12348-025-00462-0.

## Introduction

Currently, with the advancement of surgical techniques, corneal refractive surgery such as small-incision lenticule extraction (SMILE) and femtosecond laser-assisted laser in situ keratomileusis (FS-LASIK) offers benefits such as minimal invasiveness, rapid recovery, and reliable outcomes [1, 2]. Patients’ expectations for postoperative visual quality not only focus on improved vision but also increasingly emphasize postoperative comfort. Dry eye disease (DED) is a common complication leading to ocular discomforts such as dryness, gritty or foreign body sensation, itching, fluctuating blurry vision and lacrimation after refractive surgery [3, 4]. Some studies have reported that up to 95% of patients experience dry eye symptoms after corneal refractive surgery, with a 20% ~ 35% of these individuals developing chronic DE symptoms [5].

The mechanism by which refractive surgery influences ocular surface homeostasis has always been a matter of interest. A meta-analysis has reported variations in ocular surface parameters and sicca symptoms following different types of corneal refractive surgery [6]. Specifically, FS-LASIK has been associated with worse Ocular Surface Disease Index (OSDI), tear break-up time (TBUT), and corneal nerve density [7, 8]. However, other researchers have observed that dry eye indices tend to show no difference between SMILE and FS-LASIK one month postoperatively [9]. The underlying pathophysiology of DED after corneal refractive surgery is multifactorial and may be related to ocular surface desiccation, ocular surface inflammation, alteration of goblet cells (GCs), damage to the corneal nerves and corneal curvature changes [10, 11].

Many therapies have been proposed to target the underlying inflammatory mechanisms associated with dry eye. As an immunosuppressor, cyclosporine A (CsA) has been approved by the United States Food and Drug Administration (FDA) for the treatment of dry eye disease due to its ability to suppress T cell-mediated immune responses and reduce inflammation [12, 13]. Recent studies have illustrated that topical 0.05% CsA was effective in relieving early and chronic dry eye symptoms and maintaining ocular surface stability after refractive surgery [14–16]. However, the precise mechanism by which 0.05% CsA affects early postoperative ocular surface components has not been elucidated clearly.

Our study aimed to assess the early efficacy of 0.05% CsA eye drops in preserving the functionality of ocular surface components and reducing ocular surface inflammation among patients undergoing corneal refractive surgery. We evaluated the common effect of 0.05% CsA treatment in the early post-laser period based on ocular surface parameters, tear inflammatory cytokines and conjunctival cell transcription levels in patients undergoing SMILE and FS-LASIK.

## Patients and methods

This study was conducted at Shanghai General Hospital between September 2023 and March 2024 and had been approved by the ethics committee of the hospital (2022KY131). All procedures were performed in accordance with the tenets of the Declaration of Helsinki. All subjects were consented to participate in the study, and informed consents were obtained from all the subjects before examination.

### Subjects

The present study included a total of 72 subjects (144 eyes). All participants were randomized into groups receiving either 0.05% CsA eye drops treatment (CsA group) or conventional treatment (Control group), with allocation ratio of 1:1 by a computer-generated randomizer. Ultimately, 36 individuals (72 eyes) were included in each group.

The inclusion criteria for participants in this study were as follows: candidates for SMILE or FS-LASIK surgery, aged between 18 ~ 45 years, with stable refraction for 1 year. All participants had discontinued corneal contact lens wear for at least 2 weeks. Exclusion criteria consisted of individuals with a history of ocular disease, ocular surgery, ocular injury or continuous use of topical medications before refractive surgery. Patients using any other topical or systemic medications that could potentially affect the condition of the ocular surface were instructed to quit them for two weeks prior to the initiation of the study. Pregnant and nursing mothers were also excluded from this study. All tests and data analysis were performed on both eyes.

### Treatment

All baseline data and specimens were collected prior to the initiation of treatment. Preoperative management included the administration of levofloxacin eye drops and additional sodium hypromellose eye drops in cyclosporine A treated group and control group. Both groups received standard treatment according to our center’s routine postoperative therapeutic schedule during the first month after surgery. This conventional treatment included topical tobramycin eye drops six times daily for 2 weeks, 0.3% sodium hyaluronate eye drops six times daily for 1 month, 0.1% fluorometholone eye drops four times daily and reduced once every week, and hypromellose eye drops six times daily for 1 month. The CsA group received topical 0.05% cyclosporine A twice a day for 1 month additional to conventional treatment. Baseline measurements were taken within 2 weeks before surgery, and follow-up appointment was at 1 month after surgery.

### Ocular surface evaluations

The OSDI Questionnaire was used to evaluate DED-related symptoms [17]. Keratograph® 5 M (Oculus, Wetzlar, Germany) was used to examine tear meniscus height (TMH), non-invasive tear break up time (NIBUT) and photographing corneal fluorescein staining (CFS). A fluorescein strip moistened with saline solution was applied by an investigator to the inferior conjunctival fornix for CFS evaluation according to guidelines provided by the National Eye Institute Workshop (total score range: 0 ~ 15) [18].

Lipid layer thickness (LLT) was estimated by detecting interference patterns and colors from the lipid layer on the tear film using LipiView™ II (Tear Science Inc., Morrisville, NC, USA). The Schirmer’s test I (SIt) was conducted using Schirmer paper strips (5 × 35 mm) without anesthesia. The above tests were performed by a single investigator for consistency.

### Tear collection and cytokine detection

Under conditions in which the ocular surface was not stimulated (prior to commencing all ocular surface tests), 15 μL of sterile saline was gently instilled into the temporal conjunctival fornix. Using a disposable micro-capillary fluid collector, 10 μL of unstimulated tear fluid was collected from tear meniscus at external canthus within 1 min, without provoking a reflex secretion of tears. Subsequently, samples to be tested were promptly stored at −80 °C. The levels of inflammatory cytokines (IL-2, IL-4, IL-6, IL-10, TNF-α, IFN-γ and IL-17A) were detected using the human Th1/Th2/Th17 kit (BD Biosciences, USA) following the manufacturer’s protocol [19].

### Conjunctival impression and immunofluorescence

Conjunctival cells were collected from the temporal bulbar conjunctiva of each eye using a 5*5*7 mm trapezoidal nitrocellulose membrane (Pall, New York, USA) after instilling oxybuprocaine hydrochloride eye drops for topical anesthetic. The NC membrane was gently pressed against the conjunctival surface using a sterile glass rod with a rounded end under uniform pressure for 10 s. The specimens were fixed in 4% paraformaldehyde (PFA) then, followed by MUC5AC immunofluorescence staining [20].

The 4% PFA-fixed specimens were washed with 0.1% Tween-20 in phosphate buffered saline (PBS) and then blocked with 0.3% Triton X-100 and 3% bovine serum albumin (BSA) in PBS for 1 h at room temperature. Later, specimens were incubated at 4 °C overnight with 3% BSA and 0.3% Triton X-100 in PBS containing the primary antibody anti-MUC5AC (1:100; ab3649, Abcam, Cambridge, UK). Afterward, all specimens were washed using PBS and then incubated with Alexa Fluor goat Anti-Mouse IgG secondary antibody (1:100; A11029, Invitrogen) for 1 h at room temperature. Specimens were then washed and incubated with DAPI for 15 min and washed later, then mounted with fluoromout-G and were stored at 4 °C until observation. The conjunctival epithelial cells were counted as the number of nuclei per 4 mm^2^ (1024 by 1024 px) field of view. Muc5AC expression was considered as the ratio of muc5AC positive staining to the number of conjunctival epithelial cells in the corresponding visual field [21, 22].

### Conjunctival brush cytology and qPCR

The conjunctival swab was collected from both eyes in every patient with a sterile synthetic fiber swab into the lower fornix after topical anesthesia. The swab was immersed into a PBS solution, and stored at −80 °C before being tested for keratin-7 (KRT-7), Mucin 5AC (Muc5AC), interferon-γ (IFN-γ) and tumor necrosis factor (TNF-α). The qPCR primer sequences are provided in Table S1. The extraction of RNA was performed using a trace RNA extraction kit (Genstone biotech, Beijing, China), following the manufacturer’s protocol. cDNA was amplified in 20μL final volume. Quantitative real-time PCR was set up in 96 well plates using the above reagents and premix ex Taq (RR420A, Takara Bio, Beijing, China) and run on ViiA^TM^7 Real-Time PCR System.

### Statistical analyses

Statistical analyses were performed using SPSS software (version 29.0; SPSS Inc, Chicago, IL). Descriptive results are presented as mean ± standard deviation. Figures were created using the GraphPad Prism 9.5 software package. The normality assumption was checked using the Shapiro–Wilk test. Normally distributed continuous variables were compared using t-test. Non-normally distributed continuous variables were compared using Wilcoxon signed rank test. Spearman’s rank correlation was used to explore the relationship between ocular parameters and cytokines. Statistical significance was set at *P* < 0.05.

## Results

### Baseline measurements

A total of 144 eyes from 72 patients were enrolled in this study, all of whom met the inclusion criteria and completed the entire follow-up process. Baseline data were presented in Table 1. No significant differences were noted between CsA group and control group regarding demographic, ocular surface parameters or examined biomarkers before surgery (*P* > 0.05).

**Table 1:** Preoperative demographic data and ocular surface examination data of subjects

Variable	CsA Group (*N* = 36, 72 eye)	CTL Group (*N* = 36, 72 eye)	*P* value
FS-LASIK eye (%)	22 (30.6)	18 (25.0)	
SMILE eye (%)	50 (69.4)	54 (75.0)	
Gender, Male (%)	15 (41.7)	11 (30.6)	0.330
Age (y)	26.80 ± 5.80	26.50 ± 5.90	0.810
Ocular surface parameters			
OSDI	22.40 ± 15.56	20.66 ± 13.75	0.586
TMH (mm)	0.31 ± 0.10	0.30 ± 0.09	0.549
NIBUT (s)	9.40 ± 4.14	9.19 ± 4.00	0.750
LLT (nm)	57.43 ± 23.91	57.97 ± 23.00	0.887
SIt (mm)	15.93 ± 10.12	16.92 ± 9.79	0.552
CFS	2.36 ± 1.74	2.67 ± 1.66	0.178
Tear cytokine (pg/mL)			
IL-2	27.61 ± 9.47	25.73 ± 12.07	0.302
IL-4	4.92 ± 2.02	4.86 ± 2.37	0.834
IL-6	55.38 ± 25.56	61.68 ± 36.63	0.321
IL-10	7.88 ± 4.62	8.05 ± 4.78	1.000
TNF-α	5.76 ± 6.27	4.79 ± 2.71	0.748
IFN-γ	2.10 ± 0.97	2.02 ± 0.94	0.725
IL-17A	4.22 ± 3.72	4.96 ± 3.49	0.140
Conjunctival cell expression			
KRT-7	1.04 ± 0.87	0.95 ± 0.64	0.895
Muc5AC	0.85 ± 1.04	0.90 ± 0.78	0.263
IFN-γ	1.92 ± 1.45	1.73 ± 1.22	0.558
TNF-α	2.69 ± 1.51	3.11 ± 2.74	0.740

*N* number, *CsA* cyclosporine A, *CTL* control, *FS-LASIK* femtosecond laser assisted laser in situ keratomileusis, *SMILE* small-incision lenticule extraction, *y* year, *OSDI* Ocular Surface Disease Index, *TMH* tear meniscus height, *NIBUT* non-invasive tear break-up time, *LLT* lipid layer thickness, *SIt* Schirmer’s test I, *CFS* corneal fluorescein staining, *IL* interleukin, *TNF* tumor necrosis factor, *IFN* interferon, *KRT-7* keratin7, *Muc5AC* mucin5AC, *P* p value comparing CsA group and CTL group at baseline

### 0.05% CsA has protective effect on early postoperative ocular stability

**Fig. 1:**
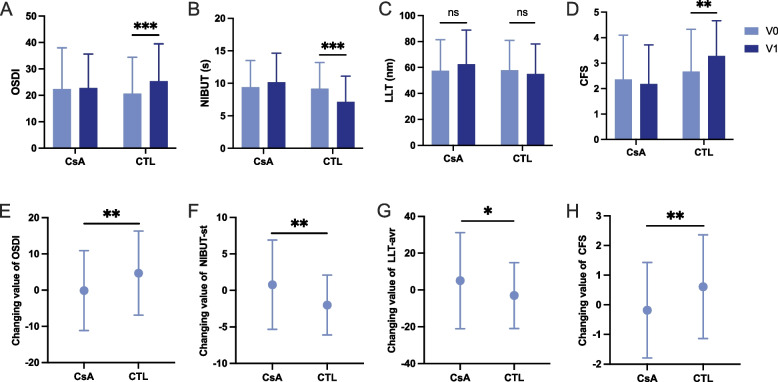
Ocular surface parameters at each time point. **A**-**D** pre- and postoperative ocular surface parameters; **E**–**H** changes of ocular surface parameters in CsA group and control group as V1 minus V0; CsA, 0.05% cyclosporine treated group; CTL, control group; OSDI: Ocular Surface Disease Index; TMH, tear meniscus height; NIBUT, non-invasive tear break-up time; LLT, lipid layer thickness; SIt, Schirmer’s test I; CFS, corneal fluorescein staining. *P < 0.05; **P < 0.01; ***P < 0.001

Figure 1 presents the values and changes in the OSDI score and ocular surface parameters between groups treated with 0.05% CsA and conventional regimen 1 month after refractive surgery. The postoperative value of OSDI and CFS score was significantly increased in control group (OSDI, 20.66 ± 13.75 vs 25.35 ± 14.17, P < 0.001; CFS, 2.67 ± 1.66 vs 3.28 ± 1.39, P = 0.004), with NIBUT significantly decreased (9.19 ± 4.00 vs 7.18 ± 3.92, *P* < 0.001) (Fig. 1A-D).

Comparing changing value between V1 and V0 (V1 minus V0) of CsA group and control group (Fig. 1. E–H), there was a more significant decline in OSDI and CFS score (OSDI, 0.41 ± 10.91 vs 4.69 ± 11.57, P† = 0.009; CFS, −0.18 ± 1.61 vs 0.61 ± 1.75, P† = 0.008), as well as rise in NIBUT and LLT after 1 month in CsA group than control group (NIBUT, 0.78 ± 6.12 vs −2.01 ± 4.10, P† = 0.002; LLT, 5.07 ± 26.09 vs −3.00 ± 17.92, P† = 0.032).

### 0.05% CsA significantly reduces level of IFN-γ and TNF-α in tear film

**Fig. 2:**
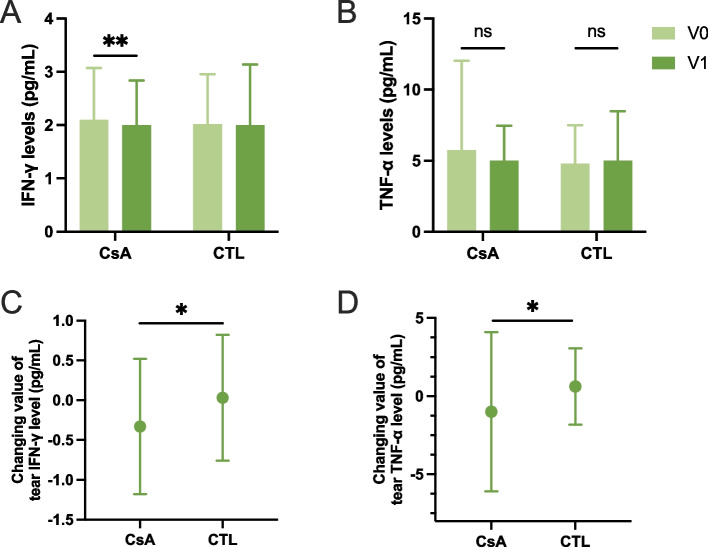
Levels of inflammatory cytokines in tear fluid at each time point. **A**, **B** pre- and postoperative levels of inflammatory cytokines in tear fluid; **C**, **D** changes of inflammatory cytokines levels in CsA group and control group as V1 minus V0; TNF, tumor necrosis factor; IFN, interferon. *P < 0.05; **P < 0.01

In tear film, the level of inflammatory cytokine IFN-γ was significantly lower after 1-month treatment with 0.05% CsA group (2.10 ± 0.97 vs 1.77 ± 0.84, P = 0.002, Fig. 2A), while TNF-α showed no significant difference in two groups before and after operation (Fig. 2B, CsA group, P = 0.243; CTL group, P = 0.053). Nevertheless, the decreasing of both IFN-γ and TNF-α levels in tear fluid was significantly greater in CsA group compared to control group (IFN-γ, −0.33 ± 0.85 vs 0.03 ± 0.79, P† = 0.012; TNF-α, −1.00 ± 5.09 vs 0.62 ± 2.45, P† = 0.032 (Fig. 2C-D)). The other inflammatory cytokines IL-2, IL-4, IL-6, IL-10, IL-17A did not change significantly after surgery under the two interventions.

### 0.05% CsA promotes maintenance of conjunctival goblet cell function after refractive surgery

**Fig. 3:**
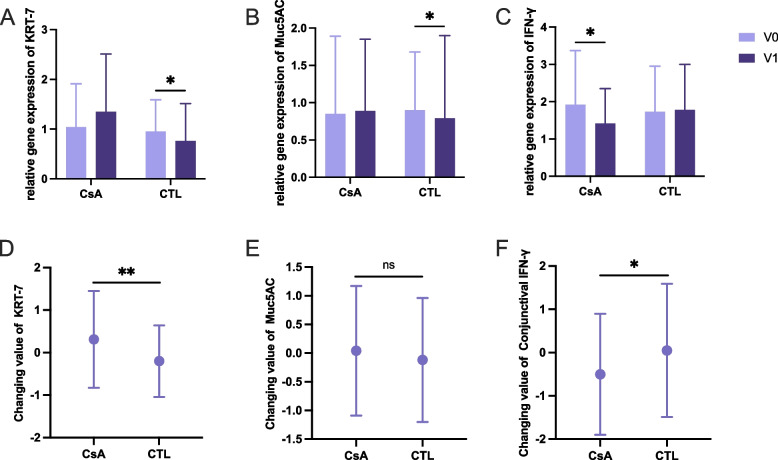
Relative gene expression of conjunctival cell related biomarkers at each time point. **A**-**C** pre- and postoperative relative gene expression of conjunctival cell related biomarkers; **D**-**F** changes of their changes in CsA group and control group as V1 minus V0; KRT-7, keratin7; Muc5AC, mucin5AC. *P < 0.05; **P < 0.01

Relative gene expression of goblet cell (GC) marker KRT-7, goblet cell-secreted mucin Muc5AC, inflammatory cytokine IFN-γ and TNF-α in conjunctival epithelial cells obtained by brush cytology was measured by quantitative real-time PCR. Levels of these factors were compared as shown in Fig. 3. In control group, KRT-7 and Muc5AC expression was lower at 1 month compared to baseline (KRT-7, 0.95 ± 0.64 vs 0.76 ± 0.75, P = 0.013; Muc5AC, 0.90 ± 0.78 vs 0.79 ± 1.11, P = 0.032. Figure 3A-B). The level of IFN-γ was significantly reduced after surgery in CsA group (1.92 ± 1.45 vs 1.42 ± 0.93, *P* = 0.013 (Fig. 3C)). There was no significant difference observed in TNF-α levels in conjunctival epithelium during the operation.

In CsA group, the increasing of KRT-7 and decreasing of IFN-γ during operation were significantly greater than that in control group (KRT-7, 0.31 ± 1.14 vs −0.20 ± 0.84, P† = 0.003; IFN-γ, −0.50 ± 1.40 vs 0.05 ± 1.54, P† = 0.019 (Fig. 3D, F)). However, the change value of Muc5AC showed no significant difference between the two groups (0.04 ± 1.13 vs −0.12 ± 1.09, P† = 0.057 (Fig. 3E)).

**Fig. 4:**
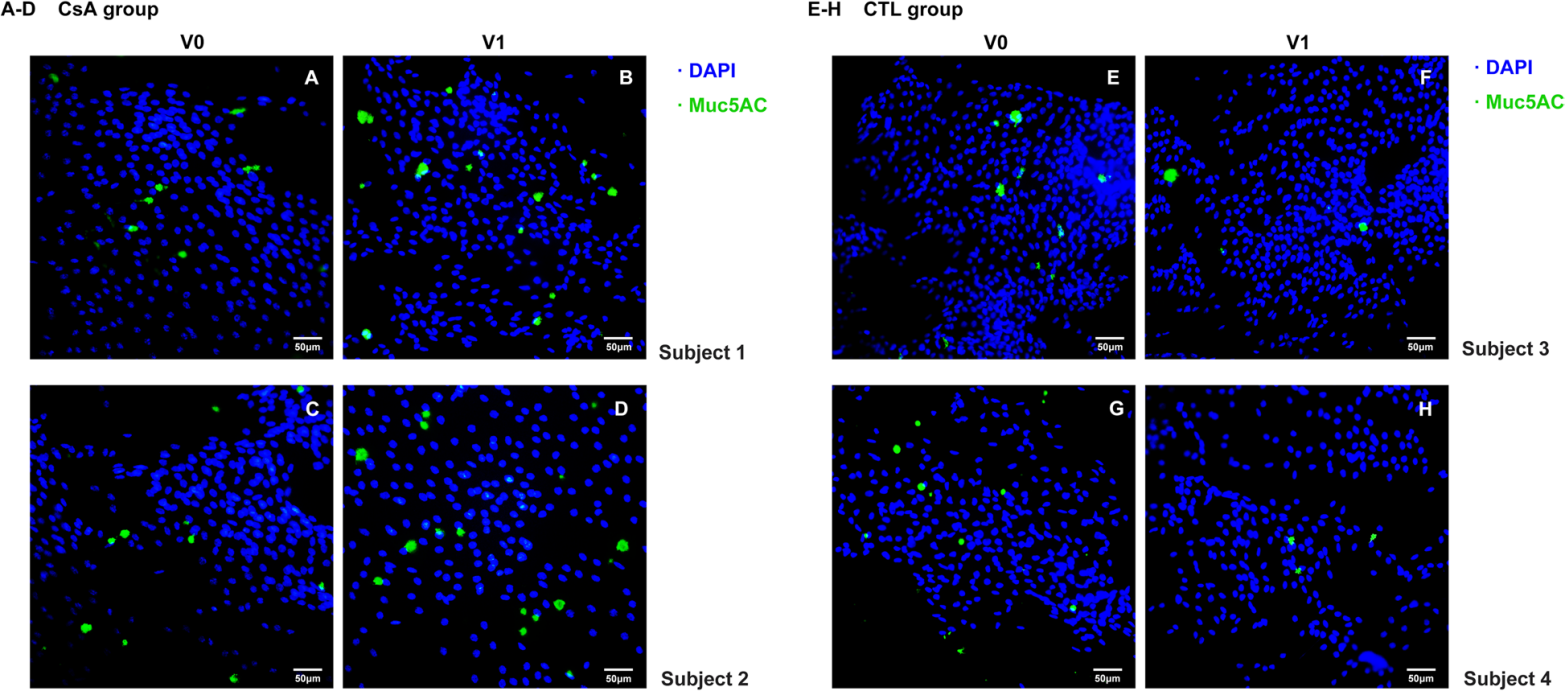
Muc5AC staining of bulbar conjunctival epithelium. **A**-**D** the IF of Muc5AC on conjunctival impression cytology at baseline and 1 month in CsA group; **E**–**H** the IF of Muc5AC on conjunctival impression cytology at baseline and 1 month in control group

We also evaluated the secretion level of Muc5AC from conjunctival epithelium by staining on impression NC membrane collected with bulbar conjunctival cells. Figure 4 illustrates the secretion of Muc5AC before and after refractive surgery. In condition of 0.05% CsA added to conventional regimen, the increase of Muc5AC expression on conjunctival epithelium can be observed in a portion of individuals (Fig. 4A-D). Conversely, the decrease was also noted in control group (Fig. 4E-F).

### Postoperative dry eye discomforts are influenced by tear inflammatory factors and GC function

**Fig. 5:**
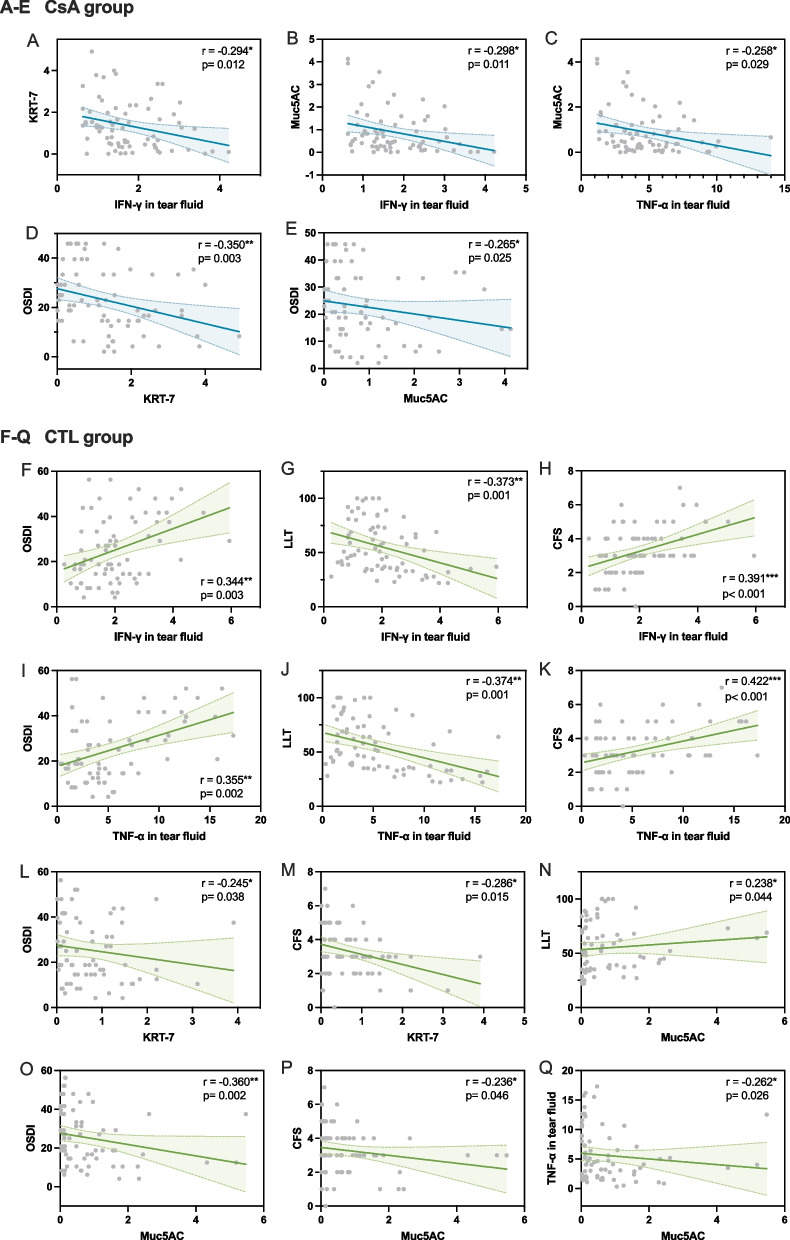
Scattergram showing the correlations between ocular surface parameters, inflammatory cytokines and goblet cells related factors in CsA and control groups at 1 month after surgery. **A**-**E** correlation of CsA group. **F**-**Q** correlation of CTL group. r, Spearman rank correlation coefficient. *p < 0.05; **p < 0.01; ***p < 0.001

Data from two groups of subjects 1 month after surgery were included for correlation analysis. In CsA group, IFN-γ and TNF-α levels in tear fluid exhibited a negative correlation with KRT-7 or Muc5AC (Fig. 5A-C). Additionally, a higher level of KRT-7 and Muc5AC was associated with decreased OSDI score (Fig. 5D-E). While in control group, the values of ocular surface parameters such as OSDI, LLT and CFS were shown to be significantly correlated with levels of IFN-γ and TNF-α in tear fluid, as well as with KRT-7 and Muc5AC (Fig. 5F-P). Consistently, TNF-α displayed a negative correlation with Muc5AC in control group (Fig. 5Q).

## Discussion

In our study, we found that the administration of 0.05% CsA treatment demonstrated a significant amelioration in postoperative ocular surface stability, tear inflammation and GC function.

After surgery, corneal wound healing occurs through promoted keratocyte apoptosis and recruitment of inflammatory cells, leading to tear film instability and disruption of corneal barrier function [15]. Our study showed that patients experienced postoperative dry eye discomforts with conventional treatment by the outcomes of the OSDI questionnaire, accompanied by shorter NIBUT and higher CFS scores. These findings suggest that tear film instability and corneal epithelial damage caused by refractive surgery may contribute to the development of dry eye symptoms.

Various topical anti-inflammatory drugs are increasingly being used adjunctively to decrease the underlying inflammation after ocular operation [11, 23]. The therapeutic efficacy of CsA on tear stability has been determined in post-LASIK patients [15]. Our research fills the gap in understanding the role of CsA in tear inflammatory cytokines and conjunctival function. According to previous research, the clinical effect of 0.05% CsA starts to be observed in the early stage of medication (2 ~ 4 weeks) [24]. Ocular surface instability and dry eye symptoms are commonly observed in the immediate postoperative period after refractive surgery [25]. Here we showed that for the subjects, CsA could alleviate tear film instability and ocular surface discomfort to a certain extent as early as 1 month after surgery. The exact mechanism of action of CsA has not been fully elucidated, a number of scientific literatures have reported its efficacy in DED immunopathophysiology, including its anti-inflammatory properties, ability to decrease GCs and epithelium apoptosis, suggesting that CsA may alter immune response on ocular surface and effectively combat inflammation, thereby controlling tear osmolarity, improving tear film stability and relieving symptoms of DED [26–28]. In this study, the aggravation of NIBUT, CFS and OSDI after surgery was reversed by 0.05% CsA treatment, supporting our perspective on the beneficial effect of 0.05% CsA on ocular surface homeostasis.

It is worth mentioning that a significantly greater increase of LLT was observed in CsA group compared to control group, suggesting a more effective impact of additional CsA on improving quality of tear lipid layer than conventional regimen alone. 0.05% CsA may reduce inflammation in meibomian glands through its highly specific immunomodulatory effects on T-lymphocytes, thus ameliorate the lipid layer thickness composed of meibum and stabilize tear film [29].

Topical anti-inflammatory medications suppress the cytokine-mediated ocular surface inflammation, improving the quality of tear film and promoting restoration of ocular surface. The downgrade of ocular inflammation gives rise to elevation of GC density, facilitating tear film recovery [11]. After SMILE or FS-LASIK surgery, there was a significant downregulation of TNF-α and IFN-γ level in tear film in CsA compared to control group. TNF-α is a pleiotropic cytokine playing a major role in coordinating inflammation and immunity [30]. Inhibition of TNF-α allowed improvement of dry eye [31]. Consistent with our results, a recent research found that artificial tears combined with CsA could downregulate the TNF-α level in tear of dry eye patients, supporting the effect of CsA on ocular surface inflammation [32]. Here we show that TNF-α exhibited a positive correlation with CFS. Several studies have emerged TNF-α as an essential regulatory factor in corneal barrier, high CFS score is related to corneal epithelial integrity [33–35]. Blocking TNF-α ameliorates the loss of corneal epithelial barrier function associated with ocular inflammation by maintaining organization of cytoskeleton [35].

IFN-γ serves as the signature cytokine produced by natural killer cell (NK), NKT cell, and activated CD4 Th1 + cells, and has been proposed as a biomarker for DED [36]. It induces apoptosis of the corneal, conjunctival (including GCs), and lacrimal gland acinar epithelium [37, 38]. At the cellular and molecular levels, the detrimental effects of IFN-γ on ocular surface are manifested through its impact on corneal stromal fibroblasts and epithelial cells, promoting inflammation, opacification, and barrier disruption [39]. In line with these concepts, we found the level of IFN-γ was related to OSDI and CFS, illustrating the damage to cornea epithelium may be associated with IFN-γ as well. Conversely, no differences observed in other dry eye-related cytokines such as IL-6 and IL-17A, this could potentially lie in inherent effect of conventional treatment responded to ocular damage.

Cyclosporine has the ability to regulate the underlying inflammatory pathology of the ocular surface by binding to cyclophilin in lymphocytes, blocking the expression of IFN-γ [40]. Previous studies have made a point that CsA could downregulated IFN-γ level in ocular disease [15, 41]. IFN-γ also promotes maturation of antigen-presenting cells (APCs) that can prime autoreactive T cells, which could be blocked by CsA [42, 43]. Conformably, our study revealed a suppression of IFN-γ levels in both tear and conjunctiva after 1 month of surgery when treated with 0.05% CsA, together with KRT-7 and Muc5AC expression also promoted in conjunctival GCs, which has not been reported previously.

According to the Dry Eye Workshop II, an appropriate number of functional GCs are required to maintain a healthy ocular surface [44]. Ocular surface inflammation and hyperosmotic stress can compromise goblet cell integrity and consequently inhibit MUC5AC expression [45]. Treatment with 0.05% CsA ophthalmic emulsion for 6 months resulted in an increase in conjunctival GC density by 191% [46]. Additionally, the upregulation of Muc5AC by CsA was also observed in dry eye patient with chronic graft-versus-host disease [47]. On the whole, the enhanced effect of CsA on GCs and mucin secretion was fully illustrated.

Moreover, our results of correlation analysis suggested relevance between IFN-γ in tear and Muc5AC as well as KRT-7 under the intervention of 0.05% CsA, which was rarely illustrated before. Previous evidence suggests that IFN-γ expression accounts for apoptosis and conjunctival GC loss [48]. In response to inflammatory stimulation, corneal and conjunctival epithelial cells as well as GCs express receptors for inflammatory cytokines such as IFN-γ, which plays a central role in inducing conjunctival metaplasia and decreasing the number of filled GCs [49, 50]. In primary cultures of mouse GCs, it has been reported that IFN-γ inhibits mucin secretion induced by cholinergic stimulus and lead to GC apoptosis and death [51]. On the contrary, IFN-γ neutralization prevented conjunctival GC loss in an experimental murine dry eye model [52]. Overall, the level of IFN-γ may take part in mediating the impact of 0.05% CsA on GC function to improve the symptoms of ocular surface dryness. While in control group, ocular surface parameters were correlated with IFN-γ, TNF-α, and GC-associated proteins, indicating that the aggravation of dry eye following conventional treatment may be related to goblet cell dysfunction and elevated levels of inflammatory mediators in tears.

It is noteworthy that our subjects included a proportion of people with large range of OSDI and NIBUT, who may be exposed to a higher risk for postoperative dry eye [53]. Therefore, early postoperative anti-inflammatory treatment is a critical strategy. Our findings extended the significance of the early effects of CsA following refractive surgery in the general population. The results from the control group indicated that OSDI, NIBUT and CFS scores worsen early after corneal refractive surgery compared to preoperative levels. Additionally, goblet cell function was downregulated in the early postoperative period, not limited to the changes in OSDI and BUT observed in healthy subjects [14, 15]. Furthermore, our study demonstrated that CsA significantly ameliorates these abnormal changes, which broadens the therapeutic applications of topical CsA in the context of corneal refractive surgery.

The current study has certain limitations. In our study sample, an imbalance was observed in the proportion of subjects undergoing FS-LASIK versus SMILE. Given that different surgical procedures affect distinct parts of the cornea, early postoperative dry eye indicators may vary accordingly. Several studies have reported differences in early postoperative dry eye symptoms and ocular surface parameters between FS-LASIK and SMILE. For instance, more severe ocular surface symptoms and BUT were noted at one month post-FS-LASIK, with greater impacts on corneal nerve fibers [6, 7, 54]. Another report suggested that the early postoperative OSDI, NIBUT, and CFS, as well as the TNF-α levels in tears showed no significantly differences between these two procedures [55]. However, research on molecular-level changes remains limited. An animal study demonstrated that inflammatory factors in the cornea and aqueous humor of rabbits recovered faster after SMILE compared to FS-LASIK [56]. Under the premise of consistent randomization and inclusion criteria, and based on the aforementioned studies, we combined subjects who underwent FS-LASIK and SMILE surgeries for the overall data analysis. We focused on evaluating the general efficacy of CsA in the early stages following corneal laser surgery, particularly concerning molecular changes in tears and conjunctiva. Future studies should explore the differences between surgical modalities regarding tear functional cytokines and the molecular mechanisms underlying conjunctival change.

The study was designed as a short-term prospective series, which may not provide sufficient evidence to fully evaluate the long-term effects of CsA treatment on ocular stability after refractive surgery. In order to enhance ocular surface recovery and prevent early postoperative dry eye deterioration, our traditional treatment regimen included a variety of effective eye drops. Therefore, our follow-up period was designed to be only one month. Furthermore, the one-month time point represents the most prominent phase of ocular surface inflammation after surgery, still, more evidence is required to support long-term results at 3 months or beyond [57]. Additionally, we tested seven specific inflammatory cytokines in this research and focused on several well-established factors for study purposes. The roles of other cytokines interacted with IFN-γ and TNF-α such as IL-13 and MMP-9 need further exploration in relation to ocular inflammatory diseases in future studies.

In summary, our research demonstrated that 0.05% CsA is effective in maintaining early ocular stability for individuals undergoing refractive surgery. 0.05% CsA alleviated dry eye related symptoms, suppressed expression of IFN-γ and TNF-α, and preserved goblet cell function as well as mucin production.

## Supplementary Information

Supplementary Material 1.

## Data Availability

No datasets were generated or analysed during the current study.

## Abbreviations

CFS: Corneal fluorescein staining
CsA: Cyclosporine A
CTL: Control
DED: Dry eye disease
FS-LASIK: Femtosecond laser assisted laser in situ keratomileusis
GC: Goblet cell
IFN: Interferon
IL: Interleukin
KRT: Keratin
LLT: Lipid layer thickness
MUC: Mucin
NIBUT: Non-invasive tear break-up time
OSDI: Ocular surface disease index
SIt: Schirmer’s test I
SMILE: Small-incision lenticule extraction
TMH: Tear meniscus height
TNF: Tumor necrosis factor
