# Supplementary material for: Efficacy of 0.05% cyclosporine A on tear inflammatory cytokines and goblet cell function after corneal refractive surgery
Source: J Ophthalmic Inflamm Infect. 2025 Apr 3;15:36. doi: 10.1186/s12348-025-00462-0 (PMC11968637; doi:10.1186/s12348-025-00462-0)
Supplement: Supplementary file 1 — Supplementary Material 1. [file 12348_2025_462_MOESM1_ESM.docx]

Table. S Primer pairs used for amplification of mRNA transcripts.

| **Gene** | **Forward (5’ to 3’)** | **Reverse (5’ to 3’)** |
| --- | --- | --- |
| **hGAPDH** | ACAACTTTGGTATCGTGGAAGG | GCCATCACGCCACAGTTTC |
| **hKRT7** | TCGAGATCGCCACCTACCGC | ATGCCACCGCCACTGCTACT |
| **hMuc5AC** | AGTGTCCCCCATGCACTGA | ACACCCTCCACAAGAAAGCG |
| **hIFN-gamma** | TGGGTTGTGTGTTTATTTCA | GCTATGTTTTCATCAGGGTC |
| **hTNF-alpha** | ACCCCCTCCTTCAGACACCC | CGAAGTGGTGGTCTTGTTGC |
